# The Cost of Dental Goods

**Published:** 1913-01-15

**Authors:** W. J. Brady

**Affiliations:** Kansas City, Mo.


### THE COST OF DENTAL GOODS.

BY W. J. BRADY, D.D.S., KANSAS CITY, MO.

It is believed by most dentists that next to diamonds and millinery, dental goods are the most expensive things to be found. It is true that dental goods cost considerable money, but most of the high-priced ones—especially those of mechanical nature—are not high-priced at all, but actually inexpensive. The real cost of an article depends on what is received for the money paid out, not just the amount it takes to buy it. Therefore we repeat that most of the expensive dental goods are cheap, even if they are costly.

There are three things that make dental goods cost money: their superlative quality, the comparatively small number of buyers, and the carelessness of dentists in both buying and paying for their supplies. But few realize the superior quality of dental goods. It is a very mild statement to say they are surpassed by very few made articles; to say they are nor equaled or even approached in quality is nearer the truth. This can only be fully appreciated by being compelled to buy and use the tools and materials offered in other lines. A real comparison is a revelation.

Compare, for instance, an automobile engine with a modern dental chair of the best make. The great exactness of the engine is loudly advertised by the manufacturer; "every part made to within one one-thousandth of an inch of exact size." Sounds like pretty fine work. Yet among good mechanics one-thousandth of an inch is considered a rather coarse measure, and in the dental chair there are parts ground to within one five-thousandth of an inch. And when it comes to hand-pieces, one ten-thousandth of an inch is all the variation allowed from standard size. There is as much machine work on a dental chair as on a four-cylinder engine, and the chair is made with greater exactness, yet the chair sells for less money than a good engine.

An ordinary screw-cutting lathe with the necessary chucks for the usual run of work costs from $90 to $125. Yet it takes less than half the work and material to build the lathe than a dental chair, and the exactness of the lathe is much the less. A precision lathe is about the only piece of machinery that ranks with a dental chair in exactness and amount of work to build; the precision lathe costs from $300 to $400, with ordinary equipment. To fit up a machine shop with precision machinery equal in variety and quantity to a well equipped dental office would cost nearly three times the money, and the quality of product would not be one particle better.

Outside of precision lathes and such machinery, the quality of dental goods is not equaled, at any price. Automatic screw machines, milling machines, and turret lathes

—all fine things—are built bigger than dental machines, but they are merely bigger and not better. The exactness of manufacture of these things does not begin to compare with chairs, dental engines, or hand-pieces. And when it comes to electric motors for dental lathes or engines, with their instantaneous stop, start, and reverse, and their silence and smooth running, and excellence and completeness of all equipment, there isn't another thing made in the entire line of electrical manufacture to be classed with dental electric goods. Such goods can't be bought elsewhere at any price, for they simply are not made.

It would naturally be supposed that jewelers and watchmakers lathes are about as exact bits of machinery as can be built. They *are* exact, but not more exact than dental hand-pieces, and many of the imported lathes not so good either in finish or temper as the ordinary hand-piece. And they wear out faster than a good hand-piece, for they are not so carefully tempered. When it comes to the thousand and one little tools that look so fascinating spread out all over the watchmaker's bench, none of them have the quality of steel nor finish of excavators or pluggers. In fact, steel goods of the temper and quality of dentists' hand instruments are not made in jewelers' and watchmakers' lines.

When it comes to burs, no such instruments are made outside of dental supplies. Small twist drills and the milling cutters for jewelers' lathes come the nearst in size and exactness of manufacture, but the quality is so inferior to a good dental bur that comparison is laughable. Yet the price is higher than for the burs. Think of getting burs with blades cut mathematically exact, each blade at a carefully calculated angle, and each exactly the same height, the edges sharp as a razor, and hard enough to cut steel, and yet so nicely tempered that breakage is almost *nil*, and the shanks all within one five-thousandth of an inch of the same size, all for a dollar a dozen. Similarly shaped cutters are made for milling machines, only larger in size—which means they are much easier to make—yet the cheapest of these is nearly a dollar apiece.

And so with many other steel goods—forceps, contouring pliers, rubber-dam punches, automatic pluggers, broaches, clamps, separators, etc.—goods of equal quality are not made outside of dental supplies. Surgical goods are a good example; surgical knives are often "pot-metal"—will not hold an edge at all; forceps bend or break on the first hard usage, saws supposed to be the finest possible are inferior to a good carpenter's saw. Example after example in numerous lines of goods might be cited, all showing inferior temper, finish, or wearing qualities, as compared with dental goods, and nearly all at higher prices.

Dental furniture is no exception to the rule. There is no work put out superior to that of the American Cabinet Company, even in the finest bank fixtures, or most exquisite house or office furniture. Compare the finest roll-top desk you ever saw with the fine dental cabinet with all its numerous smooth-sliding drawers, these with their partitions, compartments and various receptacles, all finished just as smooth and nice inside as outside. Note the amount of work put on the cabinet to make all these; see how every edge is smoothed down, every corner rounded, every place finished. Compare the wood, either solid mahogany or selected oak; the dental cabinet is the equal of the finest. Then compare the price. The dental cabinet, with equal wood, superior workmanship, and lots more of it, isn't so high after all! For any solid mahogany roll-top, or quartered-oak buffet, or even a fine dining table, will cost as much as the cabinet, and the amount of work on the cabinet is probably three times that of the other, too.

Dental goods of quality can never be sold for little money. They cost money, because it takes money to make them; and while they are expensive, they are at the same time cheap. Let us be glad that a "competition for excellence of product rather than cheapness of price"—to reverse Josiah Wedgewood's famous remark—still characterizes our best manufacturers, and we hope it will never be otherwise. And let dentists congratulate themselves that they are able to buy better things than any other profession, without exception. While the high quality of dental goods makes them costly, inferior quality makes them still more costly.

Superior quality accounts for most of the cost of dental goods, yet the limited number of buyers also helps keep the cost up. There are approximately 35,000 dentist in the United States, and about 50,000 in the world. Therefore, a dental manufacturer knows he cannot exceed this number of customers at the utmost, while many other manufacturers have the possibility of millions of customers. This limited capacity necessarily adds to the cost of dental supplies, for the laborer is always worthy of his hire. Goods must be sold at a profit, or they will not be produced.

There is another element of cost, however, that rests directly with the dentists themselves. Professional men are notoriously negligent of their financial affairs, and while dentists are no worse than the rest—and possibly not so bad as some—yet this disregard of finances makes their dental goods cost them many a pretty penny extra. This does not apply to all, of course, for there are dentists who are good business men as well as good dentists; these make a neat little saving of about thirty-three and a third per cent annually on their dental goods, all by the simple process of applying ordinary business methods to the buying and paying for the same.

It is an axiom of business that the small buyer and the slow payer always pay the long price. The cost of doing business is proportionately greater in small lots than large, all of which is and must be added to the cost of the goods. Most dentists disregard this long-established business feature entirely, and string their buying out into many small purchases where they might easily make a few large purchases and get a much better rate.

It is the quantity purchase that lowers the cost. And the quantity rate is always as advantageous to the seller as to the buyer—there wouldn't be any quantity rate unless it was. Dental dealers offer a saving of about ten per cent on really moderate quantity purchases, and this discount is certainly worth looking into. Few investments can be made to net this much and still be safe. It is safe to say no good buyer in other lines would overlook such an attractive discount, yet many dentists do, when they could just as easily bunch their orders and make money. Many a dentist grabs at a mining stock or oil well proposition that only *promises* ten per cent, and then neglects a perfectly safe thing in his own business that *pays* ten per cent.

When the deposit-in-advance method is used the cost of goods is still further reduced, and this method is just as safe as putting the money into the First National. There has never been an instance where a dentist has lost his money by this plan. It is conservatively estimated that the ordinary practitioner will "turn his money" three times a year in this way, and his savings will amount to fully thirty-three and a third per cent annually on his investment. What other proposition offers anything like this! And with safety, too.

When the extraordinary quality and limited sales of dental goods are considered, the cost is really small. And when these savings are available by the mere process of using ordinary business sagacity in buying, and following established business procedure in paying the bills, there can be no reason for howling over the cost of dental goods.—*Western Dental Journal.*

